# Enhanced Hydrofobicity of Polymers for Personal Protective Equipment Achieved by Chemical and Physical Modification

**DOI:** 10.3390/ma15010106

**Published:** 2021-12-24

**Authors:** Emilia Irzmańska, Ewa Korzeniewska, Ryszard Pawlak, Mariusz Tomczyk, Aleksandra Smejda-Krzewicka, Agnieszka Adamus-Włodarczyk

**Affiliations:** 1 Central Institute for Labour Protection, Department of Personal Protective Equipment, National Research Institute (CIOP—PIB), Wierzbowa 48 Str., 90-133 Lodz, Poland; agada@ciop.lodz.pl; 2 Institute of Electrical Engineering Systems, Lodz University of Technology, Stefanowskiego 18 Str., 90-537 Lodz, Poland; ewa.korzeniewska@p.lodz.pl (E.K.); ryszard.pawlak@p.lodz.pl (R.P.); mariusz.tomczyk@p.lodz.pl (M.T.); 3 Institute of Polymer and Dye Technology, Lodz University of Technology, Stafanowskiego 16 Str., 90-537 Lodz, Poland; aleksandra.smejda-krzewicka@p.lodz.pl

**Keywords:** protective gloves, hydrofobicity, polymer, chemical modification, laser ablation

## Abstract

The article presents significant results in research on creating superhydrophobic properties of materials which can be used as an interesting material for use in self-cleaning polymer protective gloves and similar applications where the superhydrophobicity plays a significant role. In this work the influence of laser surface modification of MVQ silicone rubber was investigated. The research was conducted using a nanosecond-pulsed laser at 1060 nm wavelength. After a process of laser ablation, the surface condition was examined using a SEM microscope and infrared spectroscopy. During the tests, the contact angle was checked both before and after the laser modification of samples pre-geometrised in the process of their production. The test results presented in the paper indicate that the chemical and physical modifications contribute to the change in the MVQ silicone rubber contact angle. A significant increase (by more than 30°) in the contact angle to 138° was observed. It was confirmed that surface geometrisation is not the only factor contributing to an increase in the contact angle of the analyzed material; other factors include a change in laser texturing parameters, such as mean beam power, pulse duration, scanning speed and pulse repetition frequency.

## 1. Introduction

Production of materials whose surface reveals hydrophobic or superhydrophobic properties is the subject of scientific research and new technological developments. The issue concerns both metallic, polymer-based materials and semiconductors [1,2,3]. The term “superhydrophobic” describes surfaces with a contact angle over 150°, which means very low wettability of such surfaces. Sample applications of such solutions include self-cleaning [4,5], anti-corrosive [6,7], anti-icing [8,9] and with reduced mechanical resistance surfaces [10]. The new approach to research on superhydrophobic surfaces was initiated by the “lotus leaf effect” described by Barthlott and Neinhus [11]. Lotus leaves owe their extremely high contact angle and related self-cleaning properties to their characteristic structure [12,13].

The contact angles of materials produced through standard technological processes typically do not exceed 120. In order to produce hydrophobic surfaces with higher contact angle values, it is necessary to carry out the material structure geometrisation and obtain a rough texture [14,15]. In materials with micro- and nanoprotrusions, the capillary forces on the air/liquid border are significantly reduced for a droplet resting on the surface. This way, a droplet can easily flow off the surface, capturing the contaminants encountered on its trajectory. Hydrophobic surfaces are characterised by low surface energy values [16]. Chemical (chemical etching, chemical deposition from the gas phase CVD, and sol-gel processes) and physical (plasma modification, laser texturing) modifications are used to obtain geometrised, superhydrophobic surfaces [6,17,18,19,20,21].

Polymer materials are commonly used in many industrial areas and contemporary engineering. The scope of their application can be broadened by improving their performance. Researchers study, for instance, the modifications aimed to achieve higher hydrophobicity of polymer materials [22,23,24].

An exciting direction of the manufacture of hydrophobic and superhydrophobic surfaces on materials focuses on the use of physical processes, including but not limited to laser texturing [25,26].

The method enables reproducible and accurate [27] formation of regular micro- and nanometric structures. The modification concept is based on using a laser beam which can introduce changes in the material structure or cause its ablation when the surface of the sample is scanned [28]. A geometrised surface formation can contribute to the increase in the contact angle of the polymer surface.

**Figure 1:**
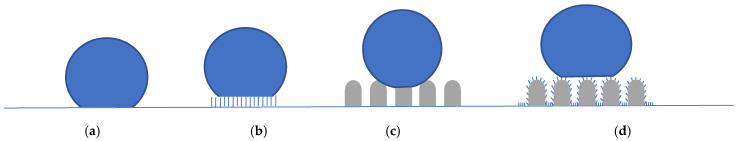
Schematic arrangement of a water droplet on (**a**) smooth and non-modified surface, (**b**) smooth and laser-modified surface, (**c**) surface geometrised during production, (**d**) hierarchic structure formed during laser modification.

During laser texturing, additional nanoprotrusions are formed on the surface of the sample. Together with the existing micrometric roughness, they form a typology typical of hierarchic structures. The process is illustrated in the Figure 1 below.

Sample laser modifications of polymer materials described in the literature aimed to improve the surface hydrophobicity and used a pulse beam of different wavelengths and various pulse durations. The wavelength of the laser beam employed in laser texturing of styrene-butadiene (SBR) and acrylonitrile-butadiene (NBR) rubber with a carbon nanotube filler was 1060 nm, and the pulse duration ranged from 15 to 220 ns. The highest contact angle value obtained was 147°. The use of picosecond pulses (70 ps) of 532 nm wavelength laser beam for the polymer surface microstructuring helped to obtain a surface morphology in the form of regularly arranged columns and the contact angle amounting to (157 ± 3°) [29]. Chen et al. modified a silicone rubber surface with 1064 nm wavelength nanosecond laser pulses of 1064 nm wavelength and obtained a surface with the contact angle of approximately 158° [30]. Zhang’s team [31] performed laser modification of silicone rubber surface using 50 nm pulse duration and 355 nm wavelength. Several repetitions of laser processing resulted in achieving the contact angle of the surface of approximately 155°. Wang et al. modified poly(methyl methacrylate) (PMMA) with a femtosecond laser (ti = 150 fs) at 775 nm wavelength, reaching the highest contact angle value of 125° [32]. Laser modification of a silicone rubber surface with a 1064 nm laser beam [33] rendered a hierarchic structure composed of densely packed nanospheres. The contact angle of the treated surface increased to 154°. Laser modifications of polymers presented above differed for the beam scanning speed, pulse generation frequency, pulse energy (mean power of the laser beam), and successive pulses and paths overlapping. It should be emphasised that identical conditions of laser texturing on different polymer materials can result in various surface topographies and contact angles [29,34,35]. The differences may result, e.g., from polymer swelling caused by gas product formation triggered by the thermal effect of the laser beam [34].

Protective clothing (personal protective equipment, PPE), including but not limited to protective gloves, constitute a vital area of polymer material application. Materials with improved hydrophobicity used in protective gloves may enhance the safety of polymer gloves users [36,37,38]. Therefore, the development of hydrophobic surfaces in such products is currently among the important research directions. Previously, standard materials used for glove production were modified by coating to improve surface hydrophobicity of the gloves [39,40]. This paper describes (chemical and physical) modifications meant to improve the hydrophobicity of silicone rubber vulcanisates, which may help extend the range of the potential applications of materials.

The performed modifications, leading to improved hydrophobicity of protective gloves, are intended to improve the safety of their use. A hydrophobic structure on the surface of protective polymer gloves facilitates the removal of contaminants deposited on their surfaces and eliminates hazardous substances outside the surface of the glove. Superhydrophobic surfaces are characterised by easy removal of contaminants and reduced risk of their contamination [41]. Moreover, a hydrophobic structure effectively reduces bacterial adhesion and diminishes the risk of penetration through the glove material [42]. Therefore, the modification of gloves to improve their hydrophobicity and provide self-cleaning properties constitutes an interesting and promising research direction.

The study used two types of silicate rubber vulcanisates: smooth (flat) and geometrised, each in two variants—non-modified and with surface modified chemically with silane derivatives. First, the contact angles of the obtained samples were measured using water. Then, laser texturing was performed on smooth and geometrised samples (non-modified and subjected to chemical modification) to achieve additional modification by forming micrometric incisions on the geometrised surface of vulcanisates. Following the physical modification, the contact angles of the samples were measured again to evaluate the influence of texturing compared to the original vulcanisates. The main purpose of the study was to determine the influence of the modifications on the change in the contact angles of silicone rubber as an interesting material for polymer self-cleaning protective gloves.

## 2. Materials and Methods

### 2.1. Preparation of Samples

Silicone rubber (MVQ) (from Chemical Plant “Silikony Polskie” Ltd., Nowa Sarzyna, Poland), dicumyl peroxide (DCP) (from Sigma Aldrich Co., Saint Louis, MO, USA) and silica (Aerosil^®^ 380) with a specific area of 380 m^2^/g (Evonik Industries AG Germany, Essen, Germany) were used for making the vulcanisates. The composition of the mixtures is summarised in Table 1.

The silicone rubber type was selected for the tests based on the current knowledge of rubber materials used to produce protective rubber gloves. The most commonly used rubber types include nitrile butadiene rubber (NBR), polyvinyl chloride and polychloroprene [43,44,45]. However, because of the study purpose, which assumed material was obtained for hydrophobic gloves production, the authors decided to extend the material search beyond the standard materials used for gloves production. Thus, silicone rubber was selected because of its properties: resistance to chemicals, good mechanical characteristics and high contact angle (about 100°) [46].

The rubber composites were prepared using a Krupp-Grusan laboratory two-roll mill with a roll diameter of 200 mm and 450 mm long. The temperature of the roll was 20–25 °C, while the speed of the front roll was 20 rpm, with the friction of the roll of 1:1.25. The ingredients were measured according to the data summarised in Table 1. In the first stage of MVQ rubber mixtures preparation, the rubber was plasticised, and then fine dicumyl peroxide and Aerosil were added. Next, the entire system was homogenised. The mixture preparation time required for thorough mixing of all ingredients was 14 min.

**Figure 2:**
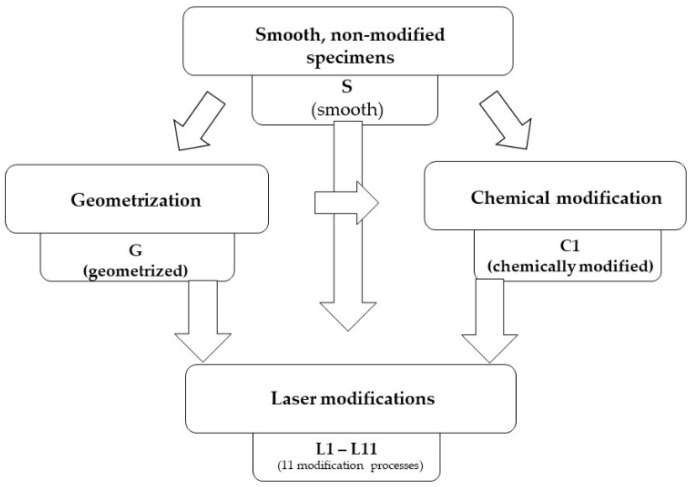
Modification procedure.

Then, the specimens were vulcanised and subjected to successive geometrical, chemical and laser modifications. Figure 2 shows a flowchart of the procedure described in this paper, including the designation of samples.

### 2.2. Geometrisation

**Figure 3:**
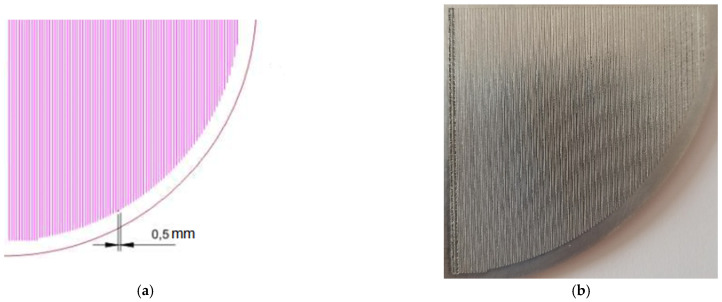
(**a**) geometrised aluminium foil design (**b**) real geometrised aluminium mould used for vulcanisation.

Rubber mixtures were vulcanised in metal moulds with an auxiliary geometrised mould to achieve geometrisation on the cross-linked silicone rubber surface. The geometrisation assumed the development of a striated structure, with the striae spacing of 0.5 mm. The mould design and its actual form are shown in Figure 3.

Rubber mixtures were vulcanised in metal moulds, between electrically heated plates of a hydraulic press. The mixture was protected with Teflon foil. A spacer (shown in Figure 3b) was placed inside the metal moulds to produce vulcanisates with a geometrised surface. The aluminium mould was made with CNS treatment methods to create a column-based structure with the pre-set distance of 0.5 mm between the striae. A 45° engraving cutter, 0.4 mm deep, was used to create the presented structure. In order to facilitate separating the geometrised vulcanisate from the mould, the previously heated spacer was sprayed with an anti-adhesive agent (McLube MAC 968, McGee Industries Inc., Aston, Anchorage, AK, USA). The rubber mixtures were vulcanised at 160 °C, under 150 bar pressure, in the time determined based on vulcametric curves. The vulcanisation time of the mixture was 10 min. The samples developed with this method are marked as G further in the paper.

### 2.3. Chemical Modification

Chemical modification of the surface with silane was carried out after the vulcanisation to improve the hydrophobicity of the obtained samples (samples marked as C1).

The surface modification involved immersing the samples in a 10% solution of n-octadecyltrimethoxysilicate in toluene for 10 min. Then, the samples were placed in an incubator at 45–50 °C and left to dry.

### 2.4. Laser Modification

**Figure 4:**
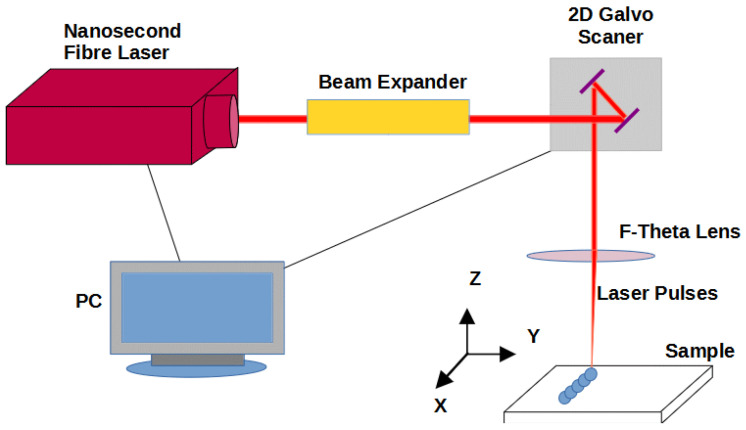
Diagram of the apparatuses used for laser modification.

Laser texturing was performed with fibre optics Nd:YAG red Energy G3 SM (SPI) laser, generating the radiation wavelength of 1062 nm. A laser beam focused with a F-theta lens (Geomatec, focal length: 160 mm) was moved along the set trajectory on the surface of sample with Xtreme (Nutfield Techn. Inc., Hudson, NH, USA) scanner. A triangulation meter (Stick, model OD2-P85W20A2) was used to establish the position of sample against the focal plane. The authors modified the surface based on their experience related to polymer surface texturing—the results were presented in a previous paper [29]. Figure 4 shows a diagram of a system for laser modification of polymer samples.

**Figure 5:**
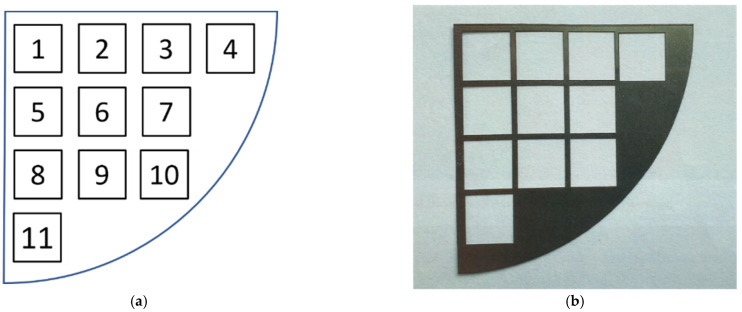
Distribution of the areas of impact of the laser beam for different variants: (**a**) arrangement of 11 test fields on the surface of the sample (**b**) the mask for locating the laser structuring areas.

Laser modification of each sample was carried out on 11 square fields, 9 mm side, arranged on the surface of the sample as shown in Figure 5a. In order to determine the influence of the process parameters on the modification results, laser texturing was performed according to the 11 variants (L1–L11) described in Table 2. In some cases, the effect of the laser beam is difficult to notice. In order to localise individual areas after laser modification, a mask was made of 150 µm thick steel foil (Figure 5b), with the geometry and dimensions corresponding to the arrangement of the 11 treatment areas (variants L1–L11, Table 2).

For the sake of the further identification of sample, the polymer plates were marked according to Table 3.

**Figure 6:**
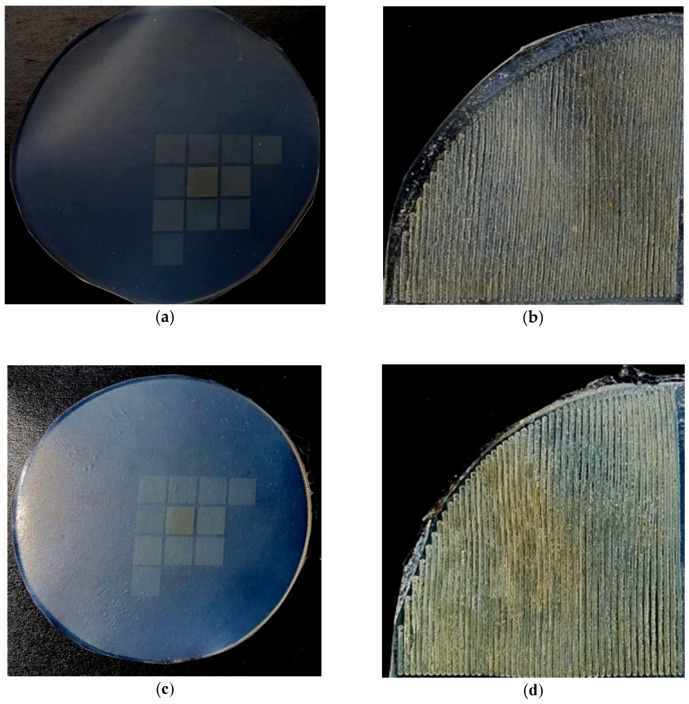
The photos of samples after laser texturing: (**a**,**b**)—MVQ silicone rubber; (**c**,**d**)—MVQ/C1 silicone rubber; (**a**,**c**)—samples with smooth surface; (**b**,**d**)—samples with geometrised surface.

Figure 6 shows the photos of the samples after laser texturing.

### 2.5. Surface Morphology Evaluation with Scanning Electron Microscopy (SEM)

The cross-section and morphology of the surface of polymer samples were examined with Tabletop Microscope TM-1000 scanning electron microscope (Hitachi, Tokyo, Japan). Before the examination, a coat of gold was vacuum-sprayed onto the samples (Sputter coater 109 auto from Cressington, 40 mbar pressure for 60 s). Then, the samples’ SEM images were analysed with TM-1000 software (Ramsay, NJ, USA) at 100 times magnification.

### 2.6. Spectra Making with the Fourier Transform Infrared (FT-IR) Method

The infrared spectra of the MVQ vulcanisates were developed with Thermo Scientific Nicolet 6700 FT-IR spectrometer equipped with Smart Orbit ATR (Waltkam, MA, USA) diamond attachment, using the attenuated total reflectance (ATR) method. The spectra were made for the wavenumber range of 3500–500 cm^−1^. Smooth and geometrised MVQ vulcanisates cleaned with acetone before and after laser modification were used in the test. Before the spectra of samples were made, background measurement had been carried out, each time including 64 scans. Identification of the absorbance bandwidth intensities helped determine the characteristic functional groups present in the tested structures of vulcanisates.

### 2.7. Contact Angle Measurements

The wettability of tested surfaces was evaluated according to the deposited droplet method using a Phoenix–Alpha apparatus from SEO (SEO, Suwon, Korea). A droplet with 10^–3^ cm^3^ volume was deposited onto the surface of sample with a syringe, and its photo was taken and analysed with Phoenix Alpha Contact Angle Analyzer software (SEO, Suwon, Korea).

The contact angle measurements for deionised water at room temperature were performed for samples before, after, and during laser texturing. For the original non-modified samples and samples after chemical modification, 10 contact angle measurements were done for each sample. For samples after laser texturing, one measurement was taken on each field corresponding to 11 variants of the process parameters.

## 3. Results

### 3.1. Evaluation of the Surface Morphology with SEM

**Figure 7:**
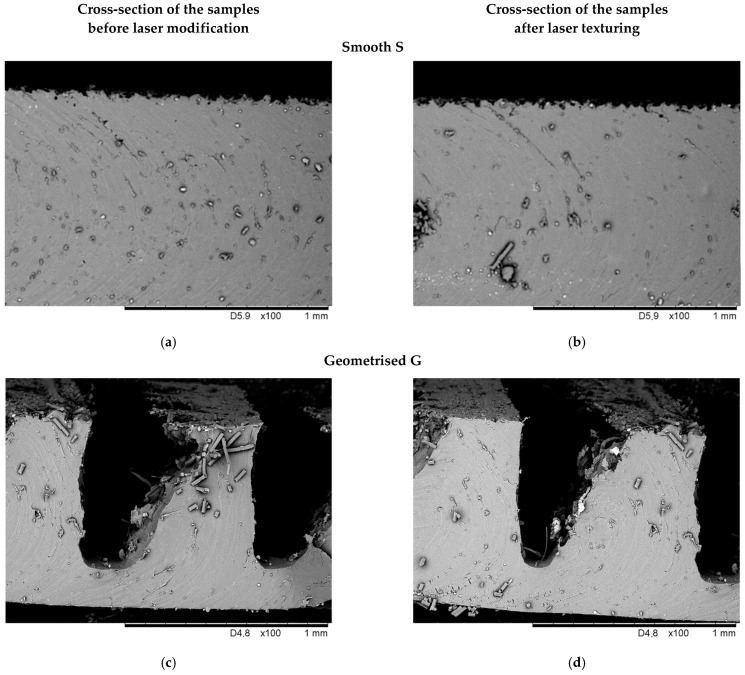
Scanning electron microscope (SEM) image of the samples’ cross-section before and after laser texturing: (**a**,**c**) cross-section of the smooth and geometrised samples before laser modification; (**b**,**d**) cross-section of the smooth and geometrised samples after laser modification.

Figure 7 presents SEM images of the samples’ cross-sections before and after laser texturing.

The results of SEM analysis, shown in Figure 7, reveal the surface of sample structure before (Figure 7a,c) and after laser texturing (Figure 7b,d). The presented images of the cross-sections of samples do not show the impact of laser texturing on the surface of either smooth or geometrised samples.

**Figure 8:**
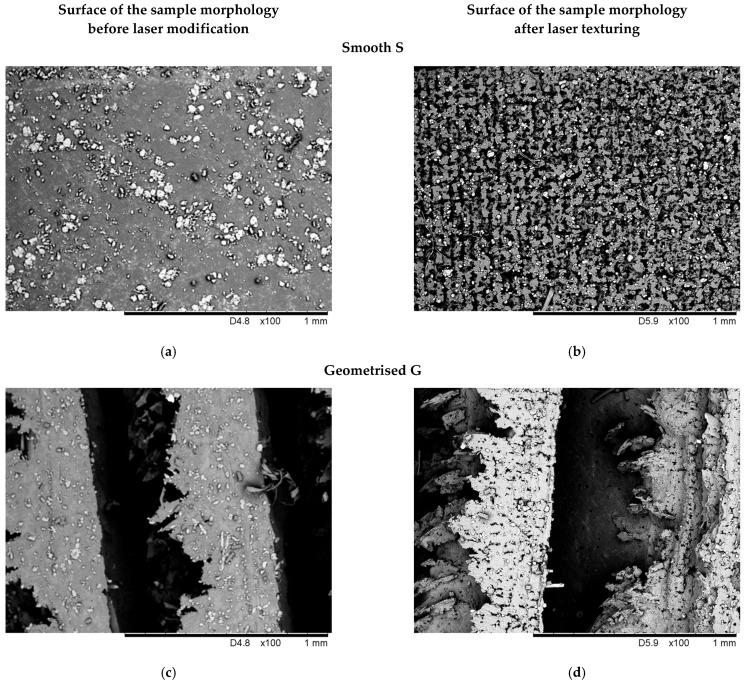
SEM image of the surface morphology of samples before and after laser texturing: (**a**,**c**) surface of the smooth and geometrised samples morphology before laser modification; (**b**,**d**) surface of the smooth and geometrised samples morphology after laser texturing.

Figure 8 shows an SEM image of the surface morphology of the sample before and after laser texturing.

The SEM examination results shown in Figure 8 reveal the difference between the structure of smooth samples (Figure 8a,c) and geometrised samples (Figure 8b,d) before and after laser texturing. In Figure 8b,d, a lumpy coat is visible, formed due to the surface cutting with a laser beam, which may increase the surface roughness but prevents water penetration into the polymer material pores. The structure of the sample after laser texturing is spongy, with irregular clusters of various sizes. The surface visualisation shown in Figure 8b,d, after laser texturing, confirms the results obtained for the tested contact angles of materials.

### 3.2. Contact Angle Measurements

**Figure 9:**
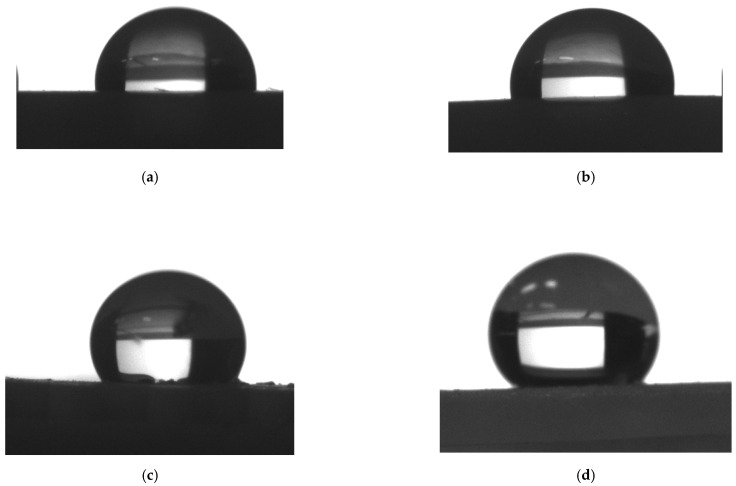
Schematic arrangement of the water droplet on (**a**) smooth, non-modified surface (S), (**b**) smooth, laser-modified surface (S-C1-L6), (**c**) surface geometrised during production (G), (**d**) hierarchic structure produced through laser modification (G-C1-L6).

Figure 9 shows photos taken during the contact angle measurements, representative of the developed samples.

**Figure 10:**
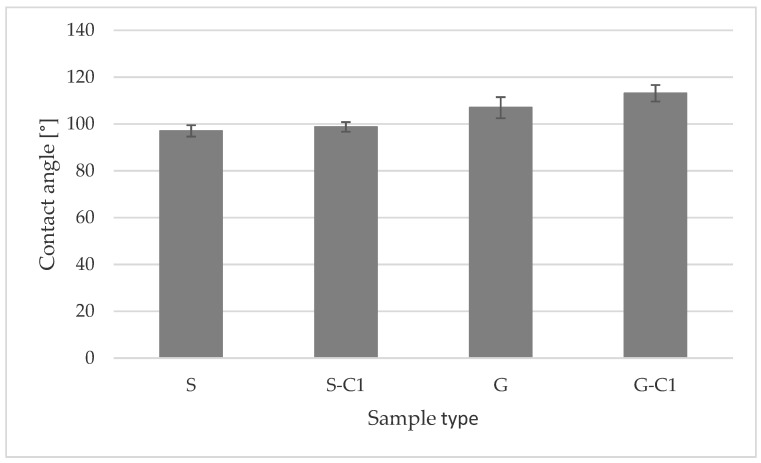
Mean contact angle values for smooth and geometrised samples, non-modified and subjected to chemical modification (S—smooth, non-modified sample, S-C1—smooth, chemically modified sample, G—geometrised, non-modified sample, G—C1 geometrised, chemically modified sample).

Table 4 and Figure 10 show the contact angle values for smooth (S) and geometrised (G) samples, non-modified and subjected to C1 chemical modification.

The mean contact angle value for a smooth sample amounted to 97.05°, whereas the contact angle of a chemically modified smooth sample was 98.74°. The surface geometrisation contributed to the contact angle increase by 10.2%, while chemical modification of a geometrised sample (G-C1) rose by 16.6% for a smooth sample (S).

Table 5 summarises the results of contact angle measurements for non-modified samples and samples after surface modification (smooth and geometrised) after laser texturing. The table includes the absolute values of the contact angle changes (Δϕ) after modification. The text continues here (Figure 2 and Table 2).

**Figure 11:**
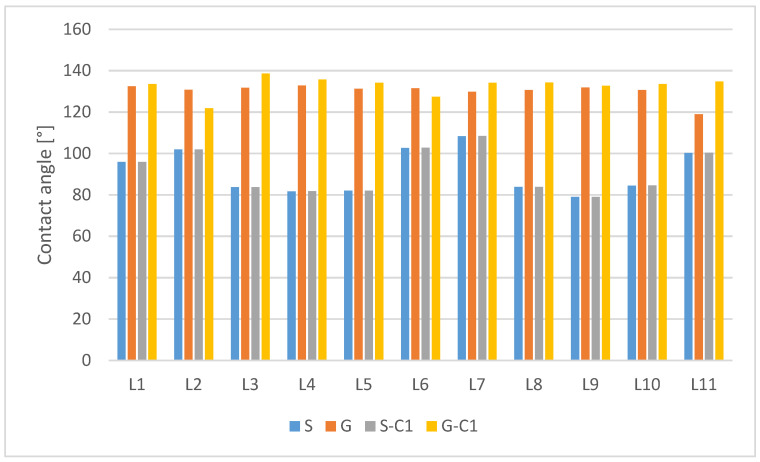
Contact angles for samples after laser texturing (S—smooth non-modified sample, S-C1—smooth, chemically modified sample, G—geometrised, non-modified sample, G-C1 geometrised, chemically modified sample).

The results obtained are summarised in the diagram (Figure 11), and a per cent change in the contact angle value is calculated for each sample in reference to the contact angle value of the non-modified sample (Table 6).

Analysing the data given in Figure 11, it can be observed that the highest values of the contact angle among the chemically non-modified samples were achieved by G geometrised samples after texturing with a laser beam with the parameters given in Table 2 for variant L4 (G-L4—132.8°). The highest increase in the contact angle value against the original smooth sample S was obtained for the G-C1-L3 sample subjected to laser texturing according to variant L3 (G-C1-L3—138.57°).

**Figure 12:**
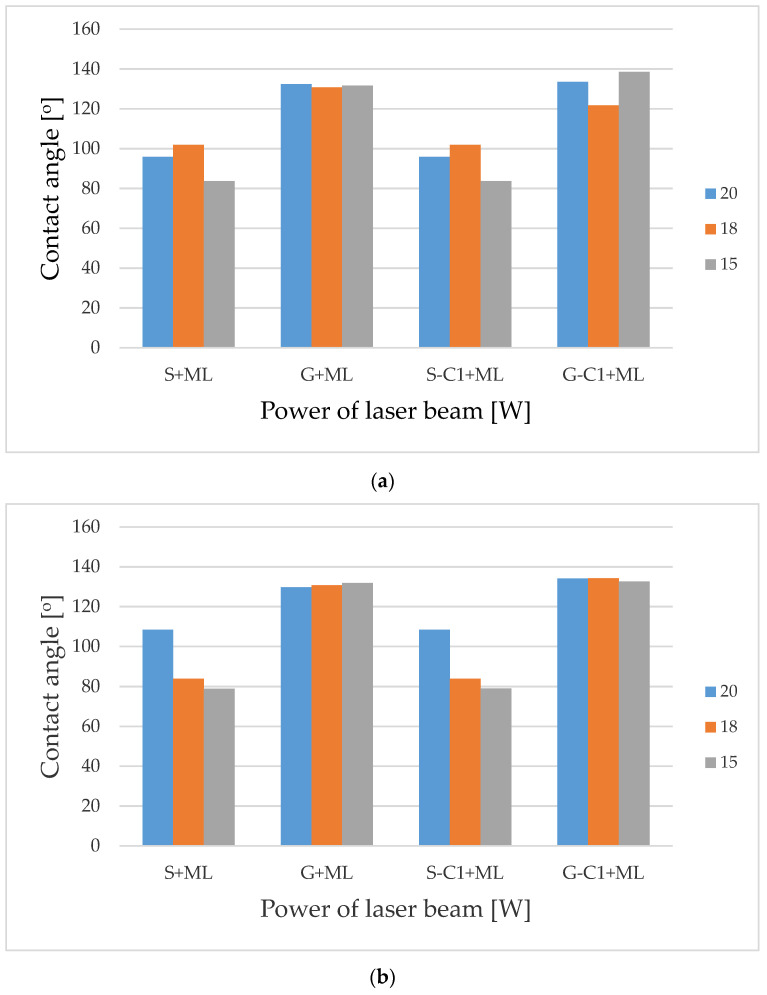
Contact angle dependence on the laser beam power function for samples after laser texturing for (**a**) t = 220 ns, v = 300 mm/s, f = 35 kHz, h = 100 µm, (**b**) t = 120 ns, v = 470 mm/s, f = 55 kHz, h = 50 µm.

For laser modification (ML), there are two main sets of laser beam parameters. In the first set, the pulse duration t = 220 ns, the scanning speed v = 300 mm/s, pulse repetition frequency f = 35 kHz and hatching h = 100 µm, while in the other set, these values amount to 120 ns, 470 mm/s, 55 kHz and 50 µm, respectively. The contact angle values in the function of laser beam power, measured for the two parameter sets, are shown in Figure 12. The plots obtained for laser modification on smooth, chemically non-modified samples (S + ML) and chemically modified samples (S-C1 + ML) nearly overlap. There are minor differences between the pre-geometrised, chemically modified and non-modified samples. For the other set of the laser beam parameters, the contact angle amounts to approximately 130° regardless of the laser power.

### 3.3. Analysis of the Vulcanisates Structure Using FT-IR Spectroscopy

**Figure 13:**
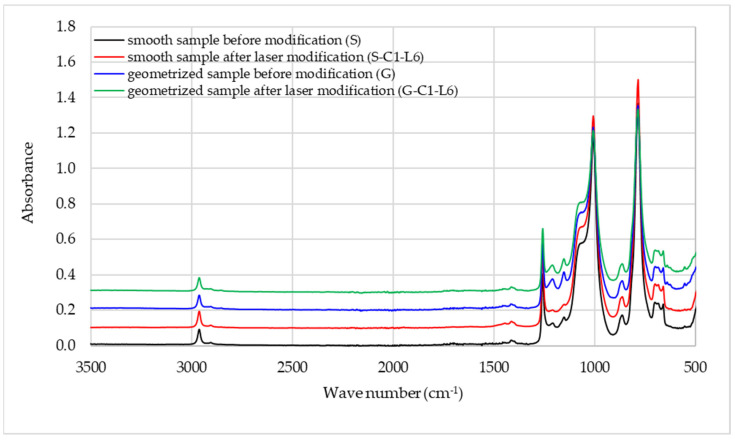
Infrared spectra of smooth and geometrised MVQ vulcanisates before and after laser texturing.

In order to evaluate laser modification impact on the structure of the examined MVQ vulcanisates, the spectra of geometrised, non-modified, and laser-textured samples were IR analysed (Figure 13). A detailed interpretation of the absorbance bands corresponding to the vibrations of characteristic functional groups occurring in the examined vulcanisate structures is presented in Table 6.

The analysis reveals the presence of two medium- and low-intensity bands located at the wave numbers of 2962 cm^−1^ and 2872 cm^−1^ for all samples’ spectra. The first band is formed due to C–H asymmetric valent vibrations of the methyl group, while the other band corresponds to C–H symmetrical valent vibrations of the methyl group. The most characteristic band for vulcanisates made of silicone rubber, observed on spectrum of each tested sample, was recorded for the wave number range of 1100–1000 cm^−1^ (intensive and broad) and at the wave number of 864 cm^−1^ (medium intensity). The bands correspond to asymmetric stretching vibrations of the Si–O group, which confirms the structure of the main chain of the MVQ macroparticle. An intensive and sharp band of deformation vibrations at the wave number of 1258 cm^−1^ is typical of the Si–CH_3_ group [47,48,49,50]. The strong and sharp band also confirms the presence of C–Si–C groups at the wavenumber of 785 cm^−1^ and a medium-intensity doublet in the wavenumber range between 700 and 660 cm^−1^. The spectrum does not reveal any significant differences in the tested samples’ spectra. The extra two absorbance bands of very low intensity (1210 cm^−1^, 1153 cm^−1^) appear only in the spectra of geometrised samples, subjected and non-subjected to laser modification. Such signals probably suggest mixed-intensity matrix vibrations between the carbon atoms in the methyl group. The fact that such vibrations are described as varied-intensity vibrations can explain their absence in the spectra of smooth samples.

## 4. Discussion

The presented studies apply to a new approach concerning the use of silicone rubber-based material (MVQ) in protective gloves. The previous solutions employed in polymer protective gloves production were based on acrylonitrile-butadiene rubber (NBR), polyvinyl chloride and natural rubber [43,44,45]. The analyses and tests performed by the authors proved that the majority of standard materials used for protective gloves production are hydrophilic. Therefore, silicone rubber (MVQ) was selected as the test material due to its hydrophobicity in the original form as well as its characteristics (resistance to selected groups of chemical substances: alcohols, thinned acids or bases; physiological harmlessness) [51,52,53]. In addition, the choice was determined by the available literature findings on the possibility to improve the hydrophobicity of silicone rubber through chemical and physical modification [30,31,33].

A significant increase in the contact angle by over 30° was observed for laser modification of pre-geometrised samples during their manufacturing. It resulted from the formation of a hierarchical structure: microroughness during the samples’ preparation and then nanoroughness due to laser ablation and local melting on the surface of the previously formed microroughness.

Laser modification turned out to be less effective for geometrised samples, additionally chemically modified, as the contact angle increased by about 20°. Laser modification of smooth, non-modified samples rendered a different result. The decrease of contact angle was observed, which means the surface of samples became less hydrophilic (comparison of samples L1, L4, L6, L7 and L10).

An analysis of laser modification parameters does not lead to any general conclusions. For geometrised samples subjected to chemical and laser modification, a two-fold hatching reduction decreases the contact angle for smooth samples. However, a minor increase in the contact angle is observed for chemically non-modified samples; the contact angle is also reduced by a low value for chemically-, and laser-modified samples (samples L1 and L6).

Reducing the laser pulse duration at a simultaneous increase in the laser beam scanning speed and the pulse repetition frequency increases the value of the contact angle. Such an effect is observed up to some optimum values of the laser beam (t = 120 ns, f = 55 kHz, v = 470 mm/s). Further modifications of the pulse duration and simultaneous increasing of the scanning speed and pulse repetition frequency change the contact angle values, but they are not optimal values. The relationships described above apply to laser modification using a 20 W power laser beam. Applying the laser treatment parameters mentioned above promotes additional roughness in the nanometric scale on the surface of the sample, whose morphology was the matrix representation. A hierarchic structure of the roughness fosters surface hydrophobicity. For a beam with a lower mean power value, the contact angle decreases. For pre-geometrised and chemically modified samples, laser modification with a 20 W beam increases the contact angle by approximately 20°, regardless of other process parameters. The results correspond to those presented in the literature. Based on SEM images, Chen et al. [30] demonstrated a hierarchical structure formed on the silicone surface after laser treatment. At lower magnifications, the surface revealed a fish scale-like structure. At higher magnifications, a spongy micro- and nanostructure was observed, where ~100 nm protrusions formed irregular clusters. The surface morphology confirmed that laser treatment contributes to the surface roughness increase and is a vital factor affecting the wettability of the surface by limiting the contact between the liquid droplets and the surface.

Rukosuyev presented a lotus leaf-like micro-nanostructure morphology of silicone rubber surface prepared with a laser. As a result of laser irradiation, two-scale hierarchical structures were formed. Sample SEM images of the textured surfaces show the influence of laser ablation on the surface, resulting in the formation of approximately 25 µm high “ridges”, composed of semi-spherical formations in a submicron scale [53].

Laser modifications described in this paper significantly influenced the change in the structure of the silicone surfaces. Irregular clusters resembling a sponge, shown in the SEM images in Figure 8b,d, contributed to the change in the modified contact angle of the samples. The best results were obtained for a geometrised sample subjected to chemical modification and L3 laser modification, for which the highest contact angle value of 138.57° was obtained. The increase of the contact angle versus the value obtained for the original smooth sample, not subjected to any modifications, amounted to 42.8%. A similar effect was obtained for a geometrised sample, subjected to chemical and L4 laser modification—the contact angle amounted to 135.72° (an increase by 39.8% versus the original non-modified, smooth sample).

The modifications did not significantly change the structures of the silicone rubber vulcanisates, which was confirmed by an IR spectra analysis. No changes in the absorbance bands of samples subjected and not subjected to laser texturing were found, which is a testimony to the fact that the laser modification described in the paper does not cause the degradation of the tested material.

## 5. Conclusions

The test results presented in the paper indicate that the performed chemical and physical modifications contribute to the change in the MVQ silicone rubber contact angle.

An analysis of the results reveals that the processes carried out did not cause a significant increase in the contact angle value for flat surfaces, and some laser modifications resulted in a significant deterioration of the samples’ hydrophobic properties (laser-textured vulcanisate L3, L4, L5, L8, L9 and L10). Identical laser modifications of geometrised samples rendered an opposite effect—a significant increase in the contact angle value (from 36% to 42%) was observed for the enumerated samples. However, surface geometrisation is not the only factor contributing to an increase in the contact angle of the analysed material; other factors include a change in laser texturing parameters, such as mean beam power, pulse duration, scanning speed and pulse repetition frequency. The best value of the contact angle was obtained for the minimum values of the abovementioned factors used in the tests (mean beam power 20 W, t = 220 ns, f = 35 kHz, v = 300 mm/s). In the future, we plan to check whether the laser modifications presented in this article may affect the degradation of the elastomer materials studied, including their degree of cross-linking and mechanical, dynamic and thermal properties. The answer to such questions would be crucial for the potential application of the proposed products. In addition, future studies will investigate the influence of chemical and physical modifications on the increase in the contact angle of silicone rubber vulcanisates.

## Figures and Tables

**Table 1:** Composition of rubber mixtures containing silicone rubber (MVQ).

Ingredient	Parts by Weight
Silicone rubber	100
Dicumyl peroxide	0.95
Aerosil 380 silica	30

**Table 2:** Parameters of the eleven variants of the silicone rubber samples modification.

Factor\Variant	L1	L2	L3	L4	L5	L6	L7	L8	L9	L10	L11
Mean power of the laser beam [W]	20	18	15	20	15	20	20	18	15	20	15
Pulse duration [ns]	220	220	220	220	220	220	120	120	120	55	55
Scanning speed [mm/s]	300	300	300	300	300	300	470	470	470	770	770
Pulse repetition frequency [kHz]	35	35	35	35	35	35	55	55	55	90	90
Hatching [µm]	100	100	100	100	100	50	50	50	50	50	50
Number of repetitions	10	10	10	4	5	4	5	5	5	5	10

**Table 3:** Designation of samples.

Before Modification	After Chemical Modification	After Laser Modification	
	C1	Without Chemical Modification	C1
S (smooth)	S-C1	S—from L1 to L11	S-C1 from L1 to L11
G (geometrised)	G-C1	G—from L1 to L11	G-C1 from L1 to L11

**Table 4:** Results of contact angle measurements [°] for smooth and geometrised samples, non-modified and subjected to chemical modification.

No.	S	S-C1	G	G-C1
1	98.8	98.4	114.7	109.5
2	99.5	98.9	102.2	108.8
3	96.2	94.8	107.6	116.2
4	94.2	99.9	112.0	114.5
5	94.1	97.1	105.9	110.3
6	94.6	102.4	110.7	118.2
7	97.3	100.3	108.6	115.8
8	99.1	97.7	102.1	116.4
9	95.9	99.9	102.0	109.3
10	100.8	98.0	104.0	112.4
Mean value	97.05	98.74	106.98	113.14
SD	2.41	2.07	4.51	3.5

**Table 5:** Results of contact angle measurements after laser texturing for chemically non-modified samples and samples after C1 modification.

			Non-Modified			After C1 Surface Modification		
Sample Number	S	Δϕ	G	Δϕ	S-C1	Δϕ	G-C1	Δϕ
L1	95.85	−1.20	132.4	33.66	95.92	−11.06	133.47	20.33
L2	101.85	4.80	130.75	32.01	101.92	−5.06	121.77	8.63
L3	83.65	−13.40	131.65	32.91	83.72	−23.26	138.57	25.43
L4	81.65	−15.40	132.8	34.06	81.72	−25.26	135.72	22.58
L5	81.95	−15.10	131.27	32.53	82.02	−24.96	134.17	21.03
L6	102.65	5.60	131.42	32.68	102.72	−4.26	127.37	14.23
L7	108.35	11.30	129.77	31.03	108.42	1.44	134.12	20.98
L8	83.75	−13.30	130.67	31.93	83.82	−23.16	134.27	21.13
L9	78.95	−18.10	131.82	33.08	79.02	−27.96	132.62	19.48
L10	84.45	−12.60	130.62	31.88	84.52	−22.46	133.52	20.28
L11	100.25	3.20	118.92	20.18	100.32	−6.66	134.67	21.53

**Table 6:** Absorption bands characteristic for smooth and geometrised MVQ vulcanisates, before and after laser texturing.

Wavenumber (cm^−1^)	Band Intensity	Chemical Group
2962	Medium	C-H in CH_3_
2872	Very weak	C-H in CH_3_
1412	Weak	CH_3_ in Si-CH_3_
1258	Intensive, sharp	Si-CH_3_
1210	Weak	C-C in CH_3_ *
1153	Weak	C-C in CH_3_ *
1100–1000	Intensive, broad	Si-O-Si
864	Medium	Si-O in O-Si-CH_3_
785	Intensive, sharp	C-Si-C
700–660	Medium doublet	Si in C-Si-C

* band present only in geometrised samples’ spectra.

## Data Availability

Not applicable.
